# Kidney Cancer Risk Associated with Historic Groundwater Trichloroethylene Contamination

**DOI:** 10.3390/ijerph19020618

**Published:** 2022-01-06

**Authors:** Angeline S. Andrew, Meifang Li, Xun Shi, Judy R. Rees, Karen M. Craver, Jonathan M. Petali

**Affiliations:** 1 Geisel School of Medicine at Dartmouth, Lebanon, NH 03756, USA; judith.r.rees@dartmouth.edu; 2 Dartmouth College, Hanover, NH 03755, USA; meifang.li@dartmouth.edu (M.L.); xun.shi@dartmouth.edu (X.S.); 3 Environmental Health Program, New Hampshire Department of Environmental Services, Concord, NH 03302, USA; karen.m.craver@des.nh.gov (K.M.C.); jonathan.m.ali@des.nh.gov (J.M.P.)

**Keywords:** renal cell carcinoma, risk factors, groundwater, residential history, solvents

## Abstract

Trichloroethylene (TCE) is a well-documented kidney carcinogen based on a substantial body of evidence including mechanistic and animal studies, as well as reports from occupational settings. However, the cancer risks for those in residential exposures such as TCE contamination in groundwater are much less clear. The objective of this study was to perform a detailed spatio-temporal analysis of estimated residential TCE exposure in New Hampshire, US. We identified kidney cancer cases (*n* = 292) and age-, gender-matched controls (*n* = 448) from the Dartmouth-Hitchcock Health System and queried a commercial financial database for address histories. We used publically available data on TCE levels in groundwater measured at contaminated sites in New Hampshire and then modeled the spatial dispersion and temporal decay. We overlaid geospatial residential locations of cases and controls with yearly maps of estimated TCE levels to estimate median exposures over the 5, 10, and 15-year epochs before diagnosis. The 50th–75th percentile of estimated residential exposure over a 15-year period was associated with increased kidney cancer risk (adjusted Odds Ratio (OR) 1.78 95% CI 1.05–3.03), compared to <50th percentile. This finding supports the need for groundwater monitoring of TCE contaminated sites to identify potential public health risks.

## 1. Introduction

The majority of primary kidney cancers (80–90%) are clear cell renal cell carcinomas (RCC), originating in the proximal tubular cells [1]. Only 2–3% of RCC cases are hereditary [1], making this disease well suited for identifying associations with environmental or lifestyle-related risk factors. Cigarette smoking is a recognized risk factor (relative risks 1.36 for current smokers, 1.16 for former smokers), and alcohol has also been implicated [2,3]. Obesity is also a risk factor, with risk correlated with increasing body mass index (BMI) prior to diagnosis [3]. Additionally, several chemical contaminants found in ambient air and certain drinking water sources have been identified as renal carcinogens, according to the US National Toxicology Program and the International Agency for Research on Cancer [4,5].

Trichloroethylene (TCE) is one such environmental contaminant that is a recognized renal carcinogen. TCE is a solvent that was commonly used to degrease metal parts, for spot cleaning laundry, as a refrigerant, as well as in certain paint strippers [6]. Human and animal studies have associated TCE exposure to renal cancer as well as non-cancer outcomes such as neurotoxicity, altered immune function, and changes in urinary markers of renal function (as reviewed by the Agency for Toxic Substance and Disease Registry [7]). Human and animal studies suggest that the carcinogenic effects of TCE are the result of metabolic biotransformation into genotoxic intermediates particularly targeting the kidney and potentially causing mutations in tumor suppressor genes therein [8]. The US National Toxicology Program lists trichloroethylene (TCE) as a “known carcinogen” in the 2016 Report on Carcinogens, citing evidence linking occupational TCE exposure to kidney cancer in 12 cohorts or nested case-control studies and 7 case-control studies [4].

Unlike occupations that directly handle TCE, residential scenarios involve chronic exposure at low to moderate doses due to vapor intrusion or private well water contamination. The various historical applications of TCE have led to several cases where improper storage, spills, or disposal cause soil and groundwater contamination. Due to its solubility and volatility, TCE exposure can readily occur from ingesting contaminated drinking water or the inhalation of vapors near the contaminated site, generating potential exposure routes for nearby residents [9].

The objective of this project was to assess the potential relationship between past trichloroethylene exposure through history of residence near contaminated sites and kidney cancer risk. We constructed yearly maps of TCE contamination based on groundwater measurements reported to the New Hampshire Department of Environmental Services and performed a detailed spatio-temporal analysis of kidney cancer risk associated with TCE exposure estimated using residential histories of cases and controls. We also used renal cell carcinoma tissue on a subset of cases to look for mutations that have been associated with TCE exposure.

## 2. Materials and Methods

*Groundwater trichloroethylene contamination:* We searched the New Hampshire Department of Environmental Services database for hazardous waste generator sites or spills containing solvents, particularly focusing on those including ‘trichloroethylene’. We queried these sites using the ‘OneStop’ online database search tool available from their website (see Data Availability) to obtain the associated laboratory reports, risk assessments, and geospatial coordinates associated with each site. We abstracted the maximum level of TCE reported in the groundwater for each site, and the year of that measurement. We also obtained data on TCE levels from hazardous waste sites identified and assessed by the US Environmental Protection Agency (US EPA) or the Centers for Disease Control/Agency for Toxic Substances and Disease Registry (CDC/ATSDR). We assumed a background TCE level of zero across the state, and placed sites with non-detectable TCE levels in this category.

From the compiled data, we used the year with the maximum contaminant level as the origin and then temporally decayed the estimate for subsequent years. Natural rates of TCE degradation in groundwater vary widely depending on the local redox conditions [10,11]. The Site Remediation Division of the Minnesota Pollution Control Agency recommends starting with a biodegradation rate assumption of −0.2 per year for their site assessments [11]. Without access to long-term natural attenuation monitoring data for New Hampshire sites that did not undergo remediation, our models used a more conservative decay rate approximating −0.12 per year.

We mapped the geospatial coordinates of each site then modeled the decaying spread of reported contaminants from that centroid point, assuming a 3000 m (1.86 mile) dispersion, based on the distance reported in the literature [12]. TCE was detected in groundwater 2.5 miles to the southeast of the former Lockformer metal-fabrication company that used TCE to degrease metal parts. As our models do not incorporate local directions of groundwater flow, we used a more conservative estimate of distance for the spatial dispersion. The levels of TCE diminish towards zero with distance from the centroid. We assigned a zero exposure value for pixels outside the spread zone. We created annual raster maps for the state with the pixel values reflecting the estimated contaminant concentrations using the snapshotting method, which was integrated into our custom ArcHealth module in ArcGIS (ESRI, West Redlands, CA, USA) [13].

*Population:* We identified kidney cancer cases and controls from within the Dartmouth-Hitchcock Health system. We restricted the current analysis to patients living in New Hampshire. Eligible cases were primary kidney cancer patients who were diagnosed 2010–2019. 90% of the cases were histologically confirmed by pathology. Controls were randomly selected primary care patients in the same healthcare system identified from among the same catchment counties as the cases during the same time period. Controls were age- and gender-matched to cases, and we omitted patients with kidney diseases from the control group.

*TCE exposure estimates*: We obtained the geocodes of addresses held by each subject over the 15-year period prior to the index date (pre-diagnostic period for cases and the equivalent time period for controls) from a commercial financial marketing database query LexisNexis (Dayton, Ohio).

To estimate the residential exposure in each year prior to diagnosis, we read the contaminant amount from the raster maps representing the dispersed contaminant for each case or control residence in each year. We then calculated the median exposure to each contaminant across his/her multiple residences in epochs representing the period prior to the index year (i.e., for the 5-year epoch of a case diagnosed in 2016, we compiled estimated exposures for residences held from 2010–2015). The Lexus Nexus search was missing addresses for less than 3 of the prior 15 years for 50% of the cases and controls (IQR 0–9 years). We chose to use the median value rather than the cumulative value to minimize bias due to these missing residences.

*Von Hippel Lindau (VHL) gene*: We selected a subset of kidney cancer cases restricting to patients with histologically confirmed renal cell carcinoma to test the hypothesis that residential exposure to TCE is associated with a VHL hotspot mutation. We identified archived formalin-fixed paraffin-embedded tissue samples on eight patients living at the time of diagnosis within a potential TCE exposure zone based on our modeling, and 24 samples from patients residing in homes without predicted TCE exposure. Tissue rolls were processed to isolate genomic DNA using the Qiagen FFPE tissue kit (Qiagen, Inc., Germantown, MD, USA). The Dartmouth Genomics and Microarray Core ran the samples on the Illumina Ampliseq ™ Cancer Hotspot Panel V2 using the Illumina Amplicon pipeline (version 2.1.1) with the hg19 genome as a reference. Call rates were >90% in 30 of the 32 samples. The mean amplicon coverage was 2213 reads, with an average of 165 variants called (range 3–636).

*Statistical analysis*: We used the residential history epoch estimates of exposure for the case-control study subjects. For each epoch of residence before diagnosis (5, 10, 15-years), the median exposure estimate of TCE was binned into categories based on the quartile distribution of each contaminant in the controls. Chi-square tests assessed the univariate difference in the proportion of cases and controls by quantile, followed by logistic regression analysis that adjusted for age, gender, alcohol use, smoking, body mass index, and diabetes. We calculated an Odds Ratio (OR) as an estimate of how much higher the odds of TCE exposure are among kidney cancer patients compared to among controls. The OR is a measure of association for a quantifying the relationship between an exposure and a disease in a case-control study, calculated using the number of kidney cancer cases who had TCE exposure, or not, and the number of controls who had or did not have this exposure [14]. These analyses were all performed using R: A Language and Environment for Statistical Computing, version 4.0.2 (R Foundation for Statistical Computing, Vienna, Austria). The Institutional Review Board of Dartmouth-Hitchcock Health approved this study.

## 3. Results

### 3.1. Population Characteristics

The residential address distribution of our study population reflects the location of Dartmouth-Hitchcock Health System facilities, with 85% of the addresses falling in western NH (Cheshire, Grafton, and Sullivan counties), 13% in south/central NH (Hillsporough, Merrimack, Rockingham, Belnap, and Strafford counties), and 2% from Carroll and Coos counties in the north. Characteristics of the study population are shown in Table 1, with a majority of males (66% of cases). Age at diagnosis was most commonly 65–75 years (34%), but 21% of cases were diagnosed before age 55. The population was largely white, non-Hispanic, reflecting the population demographics of New Hampshire.

### 3.2. Kidney Cancer Risk Factors

We obtained data to construct a multivariable model of kidney cancer risk factors established in the literature from the medical record. We found modestly increased risks associated with a body mass index (BMI) of 25–30 vs. <25 (*p* = 0.34), diabetes (*p* = 0.12), and alcohol use (*p* = 0.003). Smoking at diagnosis was associated with a statistically significant 2.43-fold increased risk of kidney cancer after adjusting for age, sex, body mass index, diabetes and alcohol (95% CI 1.72–3.43).

### 3.3. Trichloroethylene in New Hampshire

**Figure 1:**
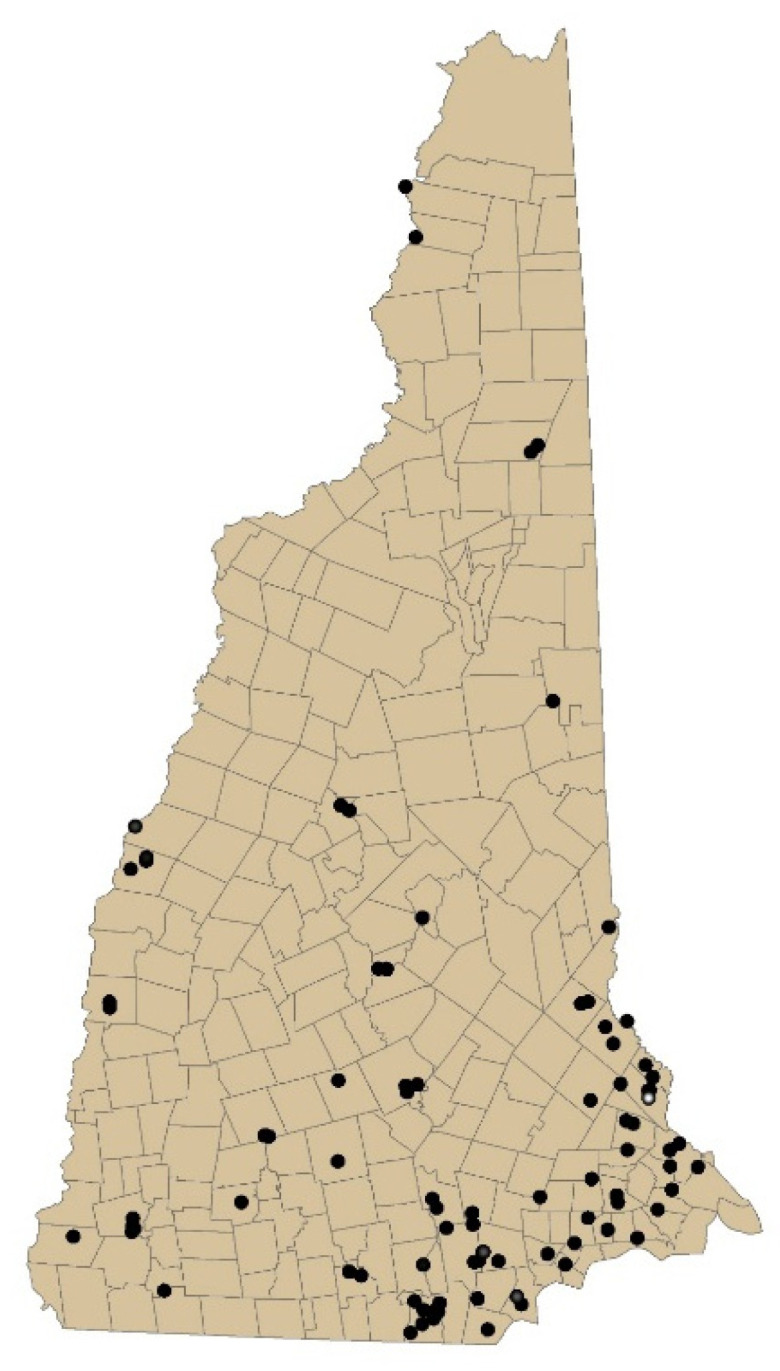
Map of New Hampshire sites containing trichloroethylene measured in groundwater.

The locations of TCE-contaminated sites in New Hampshire are shown in Figure 1. Across these 87 sites, the historic maximum measured groundwater TCE levels ranged widely (mean = 17,467, median = 135, range 2.4–760,000 ug/L). We constructed our temporally decayed and spatial spread annual model based on these site data to estimate exposures based on the geospatial locations of residence for cases and controls.

Table 2 shows the relationship between estimated trichloroethylene exposure and kidney cancer risk. Epochs 5-years, 10-years, and 15-years prior to diagnosis all show trends toward increased risk in the 50th–75th percentile of exposure, compared to exposures below the median, with a statistically significant 1.78-fold risk for the 15-year epoch. Exposure estimates diminished in the top quartile.

We also calculated the kidney cancer risk assuming a 15-year latency period between exposure and disease onset and obtained similar results (50–75th percentile OR 1.9 95%CI 1.12–3.23; 75th OR 0.94 95%CI 0.65–1.37 for the epoch 15 to 20-years prior to diagnosis) (Data Not Shown).

### 3.4. Remediation of Contaminated Sites

**Figure 2:**
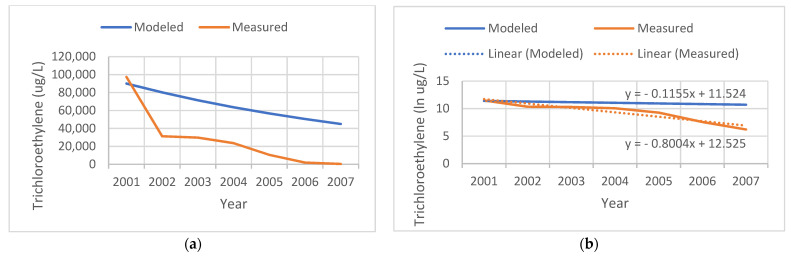
Comparison of the tricholorethylene (TCE) levels modeled (blue) versus measured (red) at a remediated site. (**a**) TCE levels in ug/L. (**b**) Natural log (ln) of TCE levels.

Figure 2 shows an example of a contaminated site that the state actively remediated. Our model assumed a linear natural decay process for TCE over the years subsequent to the initial measurement in the year 2001. Measured levels document the success of the remediation effort, with levels in 2002 dropping to 40% of those that we modeled, and measured at 500 ug/L in 2007. We modeled the slope of the temporal decay assuming a natural biodegradation process; however, due to active remediation at the site, the measured rate was faster (−0.8 per year).

### 3.5. Hotspot Mutation Assessment

**Figure 3:**
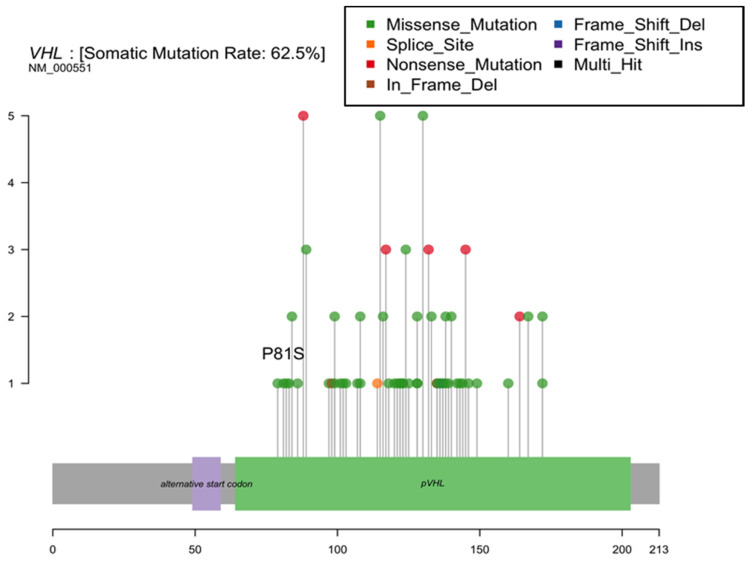
Von Hippel Lindau (VHL) gene mutations identified in New Hampshire patient renal cell carcinomas.

Based on prior reports of a TCE-linked hotspot mutation in the kidney, we assessed the hypothesis that variations in the Von Hippel Lindau (VHL) gene are associated with residential TCE exposure estimates. We observed only a single tumor with a P81S mutation at the reported hotspot (Figure 3). We found no significant increases in the proportion of tumors with any VHL mutation among patients with a history of residential TCE exposure based on our models (62.5% of those with residential TCE exposure history had a VHL mutation vs. 58.3% without exposure, *p* = 1.0).

## 4. Discussion

The objective of this study was to assess the relationship between historical residential exposure and renal cancer risk by pairing novel datasets. We used modern methods of obtaining comprehensive residential history data from a commercial financial services database for a hospital-based sample of kidney cancer cases and controls. We then performed detailed spatio-temporal modeling of TCE groundwater contamination levels to estimate possible exposures and assess risk.

Occupational data have previously documented increased kidney cancer risk with TCE exposure. A case-control study of renal cell carcinoma in France documented 86 cases and 316 controls, and those with a high cumulative dose experienced an adjusted OR = 2.16 (95% CI 1.02–4.60) [15]. Likewise, in a prospective cohort of 997 Norwegian workers, the kidney cancer Standardized Incidence Ratio was 1.7 (95% CI 1.0–3.0) [16]. A meta-analysis relative risk of occupational TCE exposure for kidney cancer was 1.32 (95% CI 1.17–1.50) [17]. While the increased risk of kidney cancer associated with occupational exposure to TCE for prolonged periods has been established, the link between residential exposure to TCE and kidney cancer has been underexplored and uncertain. An ecological study linked counties (*n* = 163) with TCE industrial discharges to higher kidney cancer mortality [18].

Several potential routes of exposure link the presence of TCE in the groundwater local to a residence and exposure to humans. Trichloroethylene exposure from contaminated water can occur through drinking, aeration, or heating of household water, and inhaling steam or skin exposure, i.e., during showering [9]. Approximately 40% of New Hampshire residents use private wells, which do not undergo mandatory testing for solvent contamination, and few residents initiate regular testing of their water source [19]. This proportion of private wells highlights the significance for direct drinking water exposure, but may contribute to overestimated historical exposures where some communities have expanded access to public water systems in response to contamination, lack of water during drought conditions or residents’ interest in accessing a regulated water supply. The EPA’s maximum contaminant level goal for drinking water is zero due to the carcinogenicity of TCE [7], but this is not an enforceable requirement for private well. The other primary route of exposure in residential scenarios is that of vapor intrusion that may occur regardless of access to a residential well or public water system [20].

TCE is a solvent that has the potential for vapor intrusion. Vapor intrusion can arise from contaminant volatilizing from localized soil contamination or from groundwater flowing underneath a home seeping into the basement air underneath a home and into the indoor air [9]. Thus, residents living near a contaminated site theoretically may inhale TCE at greater than background concentrations, even if their drinking water supply is an uncontaminated remote municipal source [21]. Contamination from local Superfund sites led to TCE levels measured at 14 μg/m^3^ in the basement air of a home in Asheville, NC, and indoor air TCE levels up to 110 μg/m^3^ in office buildings in Mountain View, CA [6,22,23]. Indoor heating during the winter months can lead to “chimney effects”, with increases in the intrusion of volatile compounds from basements into upstairs living areas [24]. Holton et al. showed substantially higher indoor air levels of TCE measured in a home above a contaminated groundwater plume winter compared to those measured in other seasons [25].

The TCE groundwater plume from the Lockformer metal-fabricating facility in Lisle, IL extended into residential wells 2.5 miles from the site point source, and the kidney cancer incidence was statistically significantly higher in the local zip code [12]. In contrast, kidney cancer incidence among residents of the neighboring region of a Superfund site in Mountain View, CA, was not significantly higher [26]. Our data in New Hampshire support an increased risk of kidney cancer with a history of residential exposure, particularly considering a longer 15-year epoch (1.78 fold risk at the 50th–75th percentile). We obtained similar results in an analysis incorporating a 15-year latency period. Since the historic groundwater levels at most sites were higher than the recent levels, it is difficult to use our study to determine biological latency.

The lack of a dose-response relationship with null effects in the top quartile for estimated exposure is potentially due to either: (1) misclassification of TCE exposure in the present study, or (2) the prioritization of exposure intervention and remediation at the most highly contaminated sites when identified. Either would plausibly explain the observed dose-response relationship.

While we had some missing historic addresses, LexisNexis address information compares well to self-reported information collected from 1099 Michigan bladder cancer study participants for a similar period of time, with 96.8% concordance between the three most recent LexisNexis addresses and those self-reported by participants [27]. A systematic comparison of residential history data accessibility, completeness, and accuracy across three different vendors identified LexisNexis to be the most effective source for residential history data when compared with survey-reported data [28].

We used a spatio-temporal approach to estimate the potential for historic TCE exposures annually statewide. However, the use of coarse assumptions for our modeling of the spread of TCE from source sites to residences is a limitation of our study. Further detailed review and analysis of additional groundwater quality data and usage history in areas surrounding the studied sites could help to refine exposure estimates, and may present an opportunity for further study. While soil TCE migrates in groundwater due to its moderate water solubility, we did not have detailed information from direct investigations of each site on the same local geology, aquifer hydrodynamics, and hydrodispersion characteristics of each plume. Under anaerobic geochemical conditions, TCE is likely to undergo reductive dechlorination to 1,2-dichloroethene [7]. While we incorporated this decay assumption in our estimation of changes in TCE levels over time, the potential for natural attenuation varies according to the specific local conditions. Additional study is needed to verify the exposure estimates or apply more refined modeling to estimate TCE dosing over the various epochs.

Our study had some additional major limitations. Although our methods aimed to quantify temporal changes in TCE exposure, we were unable to obtain comprehensive information on the remediation efforts undertaken at each of the contaminated sites, which often include delivery of bottled water to nearby residents, and engineered groundwater decontamination efforts. Further, we could not measure individual drinking water exposure, including the use of filtration and bottled water and consumption in the home and elsewhere, patterns that may have differed in areas where TCE contamination was highly publicized. As shown for the site in Figure 2, it is reassuring to observe that these efforts substantially decrease the actual measured groundwater TCE levels, and thus the potential for exposure to nearby residents. Nonetheless, this study’s lack of drinking water data is a major limitation, but historical public water supply maps were not available for our spatio-temporal analysis. Next steps would be to conduct a future study in an area with comprehensive historical maps of private wells and the sources and distribution of public water pipes, in addition to modeling dispersion and vapor intrusion from contaminated sites.

Animal studies support the link between TCE and kidney carcinogenesis. Rats experienced tubular adenocarcinoma with TCE exposure both via inhalation and ingestion [4,9]. Two-year gavage studies found increased incidences of renal tubular cell adenomas and adenocarcinomas [4]. Interestingly, Esker rats exposed to TCE (0, 100, 250, 500, 1000 mg/kg body weight by gavage, 5 days a week) for a shorter period of just 13 weeks had increases in cell proliferation in kidney tubule cells, but not preneoplastic lesions or tumors [29]. This variation in effects supports the need for testing in several models and long-term studies for accurate prediction of human carcinogenic effects, as our data also showed risks to humans over a long, 15-year exposure window. The majority of occupational exposure in humans occurs via inhalation. Rats in a chamber with TCE (50 ppm) maintained a 70% absorption fraction of the vapor over the two-hour exposure period [6]. Most of the absorbed TCE is excreted as urinary metabolites. Metabolic transformation involves glutathione conjugation to S-(1,2-dichlorovinyl)glutathione (DCVG), which forms reactive metabolites genotoxic to the proximal tubule [30]. Urinary metabolites include N-acetyl-S-dichlorovinyl-l-cysteine, and S-[2,2-dichlorovinyl] glutathione) [30].

From literature reports, occupational exposure to TCE at high levels for a prolonged period of time leads to a unique cytosine to thiamine hotspot mutation in the Von Hippel Lindau (VHL) gene resulting in a protein level change (serine for proline amino acid substitution p.P81S) [31]. In mechanistic work, the P81S change inhibits the ability of VHL to bind to hypoxia-inducible factor 1a (HIF-1a), allowing aerobic glycolysis and pushing neoangiogenesis [32]. In vitro studies confirm that genetically inducing this mutation into cells confers a pro-tumorigenic phenotype [31]. A study identified this VHL mutation in 39% of 44 RCC patients with TCE exposure, and none of the 107 unexposed RCC patients. Some of the TCE-exposed patients had multiple mutations in this VHL gene [31]. The mean time from TCE exposure onset to detection of a tumor with a VHL gene TCE-linked hotspot mutation was 23 years [31]. Another study of long-term occupational TCE exposure also identified a VHL mutation [33]. In our New Hampshire study, residential TCE exposure estimates were unrelated to VHL mutation status; this may be explained by the small sample, and the lower intensity and duration of residential exposures, compared to those historically found in occupational settings. A study of occupational TCE exposure in France also found an epidemiologic link to increased risk of renal cell carcinoma, but did not detect the mutation in the 25 exposed cases [34].

Our a spatio-temporal approach to efficiently identifying potential kidney carcinogens may be complemented by computational methods. For example, the propensity of chemicals to form DNA adducts, quantified in terms of their in silico calculated activation free energy demonstrated the carcinogenicity of a vinyl chloride metabolite [35]. This calculated prediction was validated experimentally and coincided with occupational studies linking vinyl chloride exposure to renal cell carcinoma [36]. Thus, there is also potential for a collaborative computational and spatio-temporal modeling approach to efficiently identify new potential links between contaminant exposures and disease to be investigated in the laboratory.

## 5. Conclusions

In summary, we observed an increased risk of kidney cancer associated with estimated TCE exposure based on a detailed spatio-temporal analysis of contaminated sites and residential history data. This documentation of residential risk from an environmental pollutant emphasizes the need for exposure mitigation measures to protect public health. A study of heightened cancer surveillance for members of the public with a history of TCE exposure is warranted.

## Figures and Tables

**Table 1:** New Hampshire case-control population characteristics.

		Controls	Cases	*p*-Value
	Characteristic	*N* = 448 (%)	*N* = 292 (%)	
Age	1–55	100 (22.3)	62 (21.2)	0.92
	>55–65	118 (26.3)	79 (27.1)	
	>65–75	144 (32.1)	99 (33.9)	
	>75	86 (19.2)	52 (17.8)	
Sex	Female	169 (37.7)	98 (33.6)	0.28
	Male	279 (62.3)	194 (66.4)	
Ethnicity	Hispanic or Latino/Unknown	9 (2.0)	7 (2.4)	0.63
	Not Hispanic nor Latino	439 (98.0)	283 (97.6)	
Race	Other/Unknown	8 (1.8)	6 (2.1)	0.20
	White	440 (98.2)	284 (97.9)	

**Table 2:** Estimated groundwater trichloroethylene contamination in New Hampshire and kidney cancer risk.

	Control		Controls	Cases			
Decayed TCE Exposure	Percentile	TCE (ug/L)	*N* = 448 (%)	*N* = 292 (%)	*p*-Value	OR * (95%CI)	
5-year median	<50th	0	290 (65.8)	189 (66.1)	0.14	1.0 (ref)	
	50th–75th	>0–25.3	41 (9.3)	38 (13.3)		1.47	(0.89–2.42)
	≥75th	≥25.3	110 (24.9)	59 (20.6)		0.85	(0.58–1.24)
10-year median	<50th	0	294 (66.7)	190 (66.4)	0.169	1.0 (ref)	
	50th–75th	>0–27.3	37 (8.4)	35 (12.2)		1.51	(0.90–2.54)
	≥75th	≥27.3	110 (24.9)	61 (21.3)		0.89	(0.61–1.30)
15-year median	<50th	0	298 (67.6)	189 (66.1)	0.081	1.0 (ref)	
	50th–75th	>0–27.6	33 (7.5)	35 (12.2)		1.78	(1.05–3.03)
	≥75th	≥27.6	110 (24.9)	62 (21.7)		0.92	(0.63–1.34)

* Adjusted for age, sex, alcohol, smoking, BMI, and diabetes.

## Data Availability

Contaminant data are publicly available from the Department of Environmental Services website: https://www4.des.state.nh.us/DESOnestop/BasicSearch.aspx (accessed on 6 November 2021).
